# The Role of Synthetic Data and Generative AI in Breast Imaging: Promise, Pitfalls, and Pathways Forward

**DOI:** 10.3390/diagnostics15232996

**Published:** 2025-11-25

**Authors:** Filippo Pesapane, Lucrezia D’Amelio, Luca Nicosia, Carmen Mallardi, Anna Bozzini, Lorenza Meneghetti, Gianpaolo Carrafiello, Enrico Cassano, Sonia Santicchia

**Affiliations:** 1 Breast Imaging Division, Radiology Department, IEO European Institute of Oncology IRCCS, 20141 Milan, Italy; luca.nicosia@ieo.it (L.N.); carmen.mallardi@ieo.it (C.M.); anna.bozzini@ieo.it (A.B.); lorenza.meneghetti@ieo.it (L.M.); enrico.cassano@ieo.it (E.C.); 2 Radiology Unit, Sant’Andrea University Hospital, Department of Medical Surgical Sciences and Translational Medicine, Sapienza University of Rome, 00189 Rome, Italy; 3 Radiology Department, Foundation IRCCS Ca’ Granda, Ospedale Maggiore Policlinico, Università Degli Studi di Milano, Via Festa del Perdono 7, 20122 Milan, Italy; gianpaolo.carrafiello@unimi.it (G.C.); sonia.santicchia@policlinico.mi.it (S.S.)

**Keywords:** synthetic data, generative AI, breast imaging, mammography, generative adversarial networks (GANs), diffusion models, artificial intelligence in radiology

## Abstract

Artificial intelligence is reshaping breast imaging, yet progress is constrained by data scarcity, privacy restrictions, and uneven representation. This narrative review synthesizes evidence (2020–April 2025) on synthetic data and generative AI—principally GANs and diffusion models—in mammography and related modalities. We examine how synthetic images enable data augmentation, class balancing, external validation, and simulation-based training; summarize reported gains in detection performance; and assess their potential to mitigate or, if misapplied, amplify bias across subgroups (age, density, ethnicity). We analyze threats to validity, including enriched cohorts, distribution shift, and unverifiable realism, and address medico-legal exposure, image provenance, and deepfake risks. Finally, we outline task-specific validation and reporting practices, equity auditing across density and demographics, and governance pathways aligned with EU/US regulatory expectations. Synthetic data and generative AI can enhance performance, training, and data sharing; however, responsible clinical adoption requires rigorous validation, transparency on failure modes, tamper-evident provenance, and shared accountability models.

## 1. Introduction

Artificial intelligence (AI) is rapidly transforming breast imaging, particularly in the area of screening mammography, where it has demonstrated promising capabilities in lesion detection, triage, and workflow optimization [1].

Recent prospective studies, such as the MASAI trial and ScreenTrustCAD [2,3], have underscored AI’s noninferiority compared to traditional double readings, supporting its integration into routine clinical practice.

Moreover, large-scale studies, such as the German nationwide analysis by Eisemann et al., have highlighted the potential of AI to significantly reduce radiologists’ workload while maintaining diagnostic accuracy. However, despite these promising outcomes, several challenges persist, notably in data quality, patient privacy, dataset representativeness, and equity across patient demographics [4].

A critical limitation in the current AI ecosystem in breast imaging pertains to dataset scarcity, particularly in rare pathologies or underrepresented subpopulations, and regulatory restrictions concerning patient privacy. Particularly, in breast imaging there are limited samples of young women who undergo non-routine imaging, scarcity of longitudinal imaging sequences suitable for modeling temporal evolution, and incomplete availability of paired multimodal data (e.g., mammography–ultrasound–MRI) for the same patient. Synthetic data and generative models are particularly well positioned to address these gaps by simulating rare or underrepresented scenarios and by enriching existing datasets in a controlled manner.

Synthetic data—artificially generated datasets derived from computational models—and generative AI techniques such as Generative Adversarial Networks (GANs) and diffusion models have emerged as innovative approaches to addressing these barriers (Table 1). These methodologies can create realistic and diverse mammographic images, thereby enriching training datasets, addressing class imbalances, and facilitating broader algorithm validation across heterogeneous clinical settings [5].

In this review, we use the terminology “synthetic data” to mean imaging data that are not directly acquired from a patient but are generated by an algorithm; “augmented data” denotes an expanded training set obtained by applying transformations (e.g., flipping, cropping, noise injection) and/or by adding synthetic images to real ones; and “generative data” indicates synthetic images produced by explicit generative models, such as GANs, diffusion models, or transformer-based generators. In breast imaging, most of the recent literature concerns generative data used as part of augmented datasets.

This narrative review aims to explore the role of synthetic data and generative AI within breast imaging, examining their applications, clinical implications, methodological advantages, limitations, and potential pathways forward, with the aim to give answers to the research questions that still remain open (Table 2). Emphasis will be placed on understanding how these technologies might influence clinical practice, enhance radiological education, mitigate healthcare disparities, and affect medico-legal accountability. Our synthesis incorporates literature identified via PubMed and Google Scholar searches from January 2020 to April 2025, targeting studies explicitly investigating synthetic data applications, generative AI methods, and their impacts on diagnostic accuracy, equity, and clinical workflows.

From a regulatory standpoint, it is likely that synthetic data in breast imaging will be governed primarily through existing frameworks rather than through entirely new, stand-alone regulations.

## 2. Applications of Synthetic Data in Breast Imaging

In the context of breast imaging, GANs have been utilized to synthesize mammograms exhibiting diverse tissue densities [6,7] and lesion types [8].

Breast cancer detection performance tends to be lower in high-density breasts, as dense fibroglandular tissue may obscure malignant lesions or mimic the appearance of masses. Through GAN-Driven Breast Density Transformation” introduces an innovative approach to improve the detection of hidden breast cancers in mammograms. By employing a GAN, the authors transformed high-density breast images into low-density (ACR-A) equivalents, revealing details previously obscured by dense tissue. Rather than performing a direct transformation from extremely dense (ACR-D) to entirely fatty (ACR-A) breast images, the authors implemented a progressive conversion strategy using a sequence of CycleGANs. This stepwise density reduction facilitates the emergence of previously undetectable abnormalities, especially when combined with conventional deep learning classifiers. This technique demonstrated a significant improvement in identifying malignant lesions, particularly in women with dense breast tissue [7].

Concerning lesions, in 2020, Erik Wu et al. employed a U-Net–based architecture enhanced with self-attention mechanisms and semi-supervised learning to synthesize lesions onto normal-appearing mammographic patches and to remove lesions from patches containing abnormalities. Their model successfully generated high-resolution (256 × 256 px) mammogram patches in which lesions were either inserted or removed in a realistic manner, preserving the contextual integrity of the surrounding tissue. By incorporating these synthetically augmented patches into the training process, the authors demonstrated a measurable improvement in the overall performance of breast cancer classification models. The integration of these synthetic images into the training dataset led to a significant improvement in breast cancer classification performance indeed, achieving an area under the ROC curve (AUC) of 0.846—an increase of 0.017 compared to the baseline model [8].

As a matter of fact, the synthetic images enhance the performance of deep learning algorithms in the detection and classification of breast lesions. Furthermore, synthetic breast ultrasound images have been developed, improving the robustness of classifiers in scenarios where annotated ultrasound datasets are limited. For instance, Rai HM et al. introduced the LightweightUNet model for efficient breast cancer detection. This model, characterized by low computational complexity, leveraged multimodal data from mammography and ultrasound, incorporating over 10,000 synthetic ultrasound images generated via StyleGAN3. The integration of these synthetic datasets resulted in a substantial improvement in diagnostic accuracy, increasing from 87.16% to 96.36%, thereby demonstrating the potential of synthetic data to effectively mitigate limitations inherent to real-world datasets [9].

Additionally, diffusion models, which iteratively transform stochastic noise into structured images, have garnered considerable interest due to their capacity to generate high-fidelity outputs with controllable attributes. These models have proven particularly advantageous in producing synthetic mammograms with specific characteristics such as breast density and lesion morphology, rendering them valuable tools for the development and validation of diagnostic algorithms.

The article “MAM-E: Mammographic Synthetic Image Generation with Diffusion Models” presents MAM-E, a framework composed of diffusion models designed for the synthesis of high-quality mammographic images. This system can generate images conditioned on textual descriptions and can also produce lesions localized to specific breast regions through the use of masks. The study emphasizes that conditional diffusion models represent a promising generative approach for producing mammograms with targeted features and attributes. The authors demonstrate that fine-tuning a stable diffusion (SD) model—initially pretrained on natural image datasets—using mammography data constitutes an effective method for the controlled synthesis of synthetic mammographic images. The findings indicate that their stable diffusion models achieve satisfactory performance in mammogram generation, producing visually convincing images that are challenging for radiologists to distinguish from authentic cases [10].

Another significant aspect pertains to the use of GANs to simulate “normal” breast mammograms by conditioning on images of the contralateral breast, aimed at improving the detection of occult breast carcinoma. This approach, described by Lee J et al., demonstrated that the inclusion of synthetic images during training significantly enhances the models’ ability to detect tumors not visible on standard clinical mammograms, particularly in patients with dense breast tissue. The classifier’s area under the curve (AUC) increased from 0.70 (using only real images) to 0.77 with the addition of synthetic images, suggesting a tangible benefit for early tumor detection [11].

Additionally, also the article “Perceived Realism of High-Resolution Generative Adversarial Network–derived Synthetic Mammograms” investigates the ability of GANs to generate high-resolution synthetic mammograms indistinguishable from real images. Using a dataset of over 90,000 patients, the authors trained a GAN model to produce images at 1280 × 1024 pixels. Statistical moment analysis demonstrated close alignment between the pixel intensity distributions of synthetic and real mammograms. A reader study involving 117 participants (55 radiologists and 62 non-radiologists) showed that only one participant could reliably distinguish synthetic from real images. These findings indicate that GAN-generated synthetic mammograms exhibit high perceived realism, supporting potential applications in radiology education and automated image analysis [12].

Therefore, GANs can augment existing datasets by generating high-fidelity synthetic images that closely mimic real-world data. This augmentation increases the volume and diversity of training samples, thereby improving the model’s versatility and effectiveness [13].

Another application is to use Synthetic Data for training: breast imaging education is traditionally constrained by the availability of diverse real cases and the long apprenticeship required to build expertise indeed. A recent randomized study demonstrated that radiology residents who practiced with AI-generated mammogram cases showed significantly improved cancer detection sensitivity and overall accuracy compared to those without such practice [14]. In their study, Rangharajan et al. developed a GAN to generate mammograms with adjustable characteristics (e.g., breast density, lesion size/location) and a tool allowed residents to insert simulated lesions, creating a “game-like” interactive training experience [14]. The practice group achieved measurable gains in diagnostic performance after a short training period [14], highlighting the value of synthetic images as a supplement to conventional training.

Generative AI can broaden the training curriculum by exposing trainees to a wider variety of scenarios than would be feasible with real cases alone. For example, high-resolution CycleGAN-generated mammograms have been used to translate low-density breasts into extremely dense breasts, enabling trainees to experience the challenge of detecting lesions in dense tissue [6].

By presenting the same lesion in different contexts (e.g., a subtle tumor in both fatty and dense breasts), synthetic images allow educators to assess inter-observer consistency and help trainees learn how varying tissue characteristics affect visibility [6].

This adaptability means simulation curricula can be personalized to target each trainee’s weaknesses—such as focusing on cases with very dense breasts or uncommon tumor presentations—in a risk-free environment.

In practical terms, integrating synthetic data into radiology training could involve tiered difficulty levels or competency-based progression. For instance, an interactive simulator might first present straightforward synthetic cases and gradually introduce more challenging ones (subtle lesions, post-surgical changes, etc.) as the trainee’s skills improve. Key advantages of generative training data include:

- Volume and variety: Unlimited cases covering diverse anatomies and pathologies (including rare cancers or artifacts) can be generated, overcoming scarcity of teaching files;
- Controlled complexity: Image parameters (noise, density, lesion conspicuity) can be tuned to adjust case difficulty in a systematic way;
- Immediate feedback: Trainees can receive instant feedback on AI-generated cases, enabling rapid iteration and deliberate practice.

Early evidence suggests that such simulation-based education has tangible benefits for learning outcomes [14]. Moving forward, collaborations between academic radiology departments and AI developers are needed to refine the realism of synthetic mammograms and ensure they encompass the spectrum of true clinical variability. Ultimately, incorporating generative AI into radiologist training could accelerate the learning curve in mammography, better preparing trainees to interpret the full breadth of breast imaging appearances encountered in practice [14]. By augmenting traditional apprenticeship with “virtual flight simulator” experiences, synthetic data may help produce more proficient breast radiologists in less time [14].

In conclusion, the use of GANs in healthcare for purposes like data augmentation, dataset enrichment, and balancing is highly beneficial and reliable [15].

Beyond dataset augmentation, synthetic data are also critical for algorithm validation. Controlled variations in image characteristics allow researchers to systematically evaluate model performance across diverse clinical scenarios. Qixin H et al., in their study “Synthetic Data as Validation”, emphasize the role of synthetic data in enhancing the validation process of AI models, particularly in medical imaging, where data scarcity and variability are prevalent. The authors demonstrate that synthetic data can diversify validation sets, thereby improving model robustness and performance across different clinical scenarios [16]. Although this study does not specifically address breast radiology, the demonstrated effectiveness of synthetic data in mitigating overfitting and supporting early cancer detection invites consideration of its potential application to mammographic screening and other breast imaging tasks. Such validation is essential prior to clinical deployment, especially in sensitive applications such as screening. The rigorous methodology employed by Lang et al. in the MASAI trial provides a valuable example, and similar strategies could be applied to synthetic data to ensure clinical reliability [2].

## 3. Benefits of Synthetic Data and Generative AI

Generative AI is a category of machine learning models that create data or outputs closely mimicking real-world examples. It represents a significant progression in predictive modeling approaches for breast cancer detection [5]. Generative AI supports breast cancer detection by producing realistic synthetic mammograms that enhance training datasets and improve model generalization across populations and imaging conditions. It also enables effective data augmentation through varied image generation, strengthening model robustness. Additionally, it helps reduce bias by creating more balanced datasets that reflect diverse demographic and biological characteristics [5]. Moreover, the acquisition and annotation of medical imaging data involves substantial costs. Generative AI helps reduce these expenses by generating synthetic images, which are significantly more cost-effective and quicker to produce than collecting real-world data [5].

Concerning the benefits of Synthetic Data, one of the primary advantages is the ability to overcome restrictions related to patient data privacy. Given the sensitive nature of medical data, anonymization is essential to safeguard patient confidentiality. All identifiable information must be removed in accordance with regulations such as HIPAA in the United States and GDPR in Europe [5]. Since synthetic images do not correspond to real individuals, they can be freely shared among research institutions, thereby facilitating multicenter collaborative studies. This aspect is particularly relevant in regulatory environments governed by stringent policies, such as the European Union’s General Data Protection Regulation (GDPR).

In terms of efficiency, synthetic data reduces the time and costs associated with manual annotation and expert review. Once a generative model is trained, it is possible to produce large volumes of annotated images, thereby accelerating the iterative cycles of training and validation [17].

Synthetic data also enhances dataset diversity, a critical factor for developing truly generalizable AI tools [15]. It is possible to generate images representative of underrepresented populations or simulate rare lesion types [8]. This approach contributes to the development of more equitable AI that balances efficiency, personalization, and fairness in screening programs [1].

Notably, AI algorithms in breast imaging must be developed and deployed with an eye toward health equity, as biases in training data can translate into unequal performance across patient populations. Representation bias arises when certain demographic groups (e.g., racial minorities, younger patients, those from low-resource regions) are underrepresented in the data used to train models [18]. This “health data poverty” means AI tools may generalize poorly to these groups, potentially exacerbating healthcare disparities [19,20]. For example, if a mammography AI is primarily trained on images from Caucasian women in high-income countries, its accuracy might falter on patients of different ethnicities or from underserved communities. Indeed, a recent analysis of AI in mammography highlighted geographic and population imbalances in research data, raising concerns that current models may not equitably serve diverse populations (e.g., limited representation of patients from Africa or Latin America) [21,22]. As Talby aptly noted, “Generative algorithms are only as good as the data they are trained on, and any bias in the source data will be reflected in the generated synthetic data” [21]. In other words, if the real-world data are skewed, synthetic data derived from them will likely perpetuate those biases [23].

On the other hand, generative AI offers new tools to mitigate bias if used thoughtfully. By creating synthetic examples of underrepresented groups, one can augment datasets to be more balanced. A compelling demonstration comes from Marchesi et al. (2025) [19], who developed a conditional GAN to generate synthetic health records focused on minority subgroups. They reported that augmenting training data with these synthetic minority samples improved model fairness and predictive performance for Black patients and female patients in their evaluation [19]. In the imaging domain, generative augmentation could analogously be used to boost representation of, say, women under 40 (who have fewer mammograms available) or patients with rarer tumor subtypes. Another study addressing breast density—a factor associated with both cancer risk and imaging difficulty—used CycleGANs to generate mammograms with higher density to counter class imbalance. The authors found this approach helped improve a mass detection model’s generalization to very dense breasts [6]. Notably, they emphasize that fairness of AI for women with extremely dense breasts (often younger or genetically predisposed patients) needs improvement, and synthetic data is a viable strategy to achieve that [24].

Ensuring equitable AI in breast imaging will require a multifaceted approach [22]. Data diversity should be a key performance metric—models should be tested across subgroups (different ethnicities, ages, breast densities, etc.) to identify bias [25]. Generative data can then fill gaps: for instance, creating synthetic mammograms emulating imaging from underserved populations or simulating rare pathologies primarily seen in certain groups. However, rigorous validation is crucial; synthetic augmentation is not a panacea for bias. Care must be taken that the synthetic cases are realistic and do not introduce spurious features that could mislead algorithms. Efforts are underway to develop “fairness metrics” for synthetic data [21] and to devise techniques (e.g., debiasing pipelines) that ensure generated datasets truly enhance representation without replicating prejudicial patterns [17]. In clinical practice, the implications of equitable generative AI are significant: if successful, these methods could help AI tools maintain accuracy for traditionally underserved groups (such as minority women, rural populations, or others often missing from clinical trials and imaging datasets) [22,23,24,25]. This would promote more consistent diagnostic performance across patient demographics, aligning AI deployment with goals of health equity. Conversely, neglecting this issue risks “baking in” existing disparities—a pitfall that the field must consciously avoid [25]. Thus, the pathway forward involves using synthetic data as a force-multiplier for diversity, guided by continuous monitoring and inclusion of stakeholders from underrepresented communities in the AI development process [26].

## 4. Generative AI and Medico-Legal Accountability in Breast Imaging

The introduction of generative AI into breast imaging raises complex medico-legal questions regarding responsibility and trust [17,23,24,25,26,27,28]. In the traditional radiologist-patient relationship, the radiologist bears duty of care and is legally accountable for diagnostic decisions. If an AI (for example, an autonomous screening mammogram reader or an image enhancement tool) errs—by missing a cancer or prompting an unnecessary biopsy—who is liable for the outcome? Potentially culpable parties include the interpreting radiologist who used the AI, the hospital or clinic that deployed it, and the software manufacturer or developer of the AI system [17,23,24,25,26,27,28,29]. The lines of accountability become blurred when an algorithm is involved in decision-making [26]. Early guidance from some regulators suggests holding clinicians responsible for AI’s mistakes as if they were their own. For instance, the Federation of State Medical Boards in the U.S. indicated in 2024 that physicians, not AI companies, should be answerable if an AI error leads to patient harm [30]. The rationale is that current AI tools are typically “assistive”—the final interpretation still ostensibly lies with the radiologist, who should exercise independent judgment [31]. Under this paradigm, failing to recognize an AI’s mistake would be seen as a medical error by the physician.

However, as AI systems evolve toward greater autonomy, there is a counterargument that liability should shift (at least partially) to the AI creators. Authors have even suggested requiring AI algorithms to carry malpractice insurance or legal “personhood” in certain contexts [32]. This would treat a highly autonomous AI like a drug or device that can malfunction, making the company responsible for defects. In breast imaging, this debate is more than theoretical: AI-based screening programs are being piloted, and questions arise such as whether a false-negative AI screening (missing a tumor that a human might have caught) is a case of radiologist negligence or a faulty product. Ultimately, a shared liability model may emerge, where responsibility is apportioned based on how the AI was used and the level of oversight. Factors likely to influence legal outcomes include the algorithm’s FDA approval status, the transparency of its recommendations, and whether the clinician adhered to standard of care in integrating the AI’s input [33].

In addition to medico-legal liability, draft standards now provide more concrete guidance. The 2024 ESGAR consensus statement emphasized the necessity of task-specific validation of synthetic data, recommending that studies include both radiologist reader studies and algorithmic benchmarking across representative populations [34,35]. Incorporating guidelines like this anchors medico-legal and ethical discussions in the emerging regulatory landscape.

Beyond malpractice concerns, generative AI challenges the integrity of medical images and records, raising new legal and ethical issues. Deepfakes—highly realistic altered images—have made their way into healthcare. Researchers have demonstrated the ease of using GANs to inject or remove lesions in medical images, producing convincing fakes that could mislead diagnosis [36]. In breast imaging, one can imagine nefarious scenarios (e.g., someone alters a mammogram to simulate a cancer for fraudulent purposes, or conversely removes a cancerous lesion to cover up negligence). Such possibilities erode trust in the authenticity of imaging data. If a clinical decision is based on a falsified image, liability could extend to those who failed to maintain secure image provenance. Legal frameworks will need to catch up to punish malicious creation or use of deepfake medical images, akin to how laws are developing for deepfakes in other domains. Proactive measures are being called for to ensure the veracity of medical images—for example, using cryptographic image signatures or blockchain to detect tampering [37]. The healthcare community should implement policies and technical safeguards before trust is eroded by undetected deepfakes: from a medico-legal standpoint, institutions might need to demonstrate due diligence in guarding against altered or synthetic data entering clinical workflows.

A further medico-legal and privacy concern relates to model memorization, whereby a generative model may inadvertently reproduce images that are too close to specific training cases, potentially enabling re-identification. Technical safeguards include regularization and early stopping to reduce overfitting, explicit de-duplication checks comparing synthetic outputs to the training corpus, and, in some settings, the use of differential-privacy techniques during training. From a governance perspective, institutions should avoid training generative models on very small, highly identifiable cohorts and should document procedures used to verify that released synthetic images are not near-exact replicas of real patient data.

In summary, generative AI in breast imaging holds great promise but also brings new accountability challenges. Radiologists may need updated guidelines on how to incorporate AI outputs into their medical decision-making while maintaining professional responsibility. Hospitals and regulators will likewise need to establish clear protocols for quality control, error disclosure, and liability when AI is in the loop. As one commentary noted, the ultimate role of AI in radiology will be heavily influenced by how legal liability for errors is resolved [38]. Striking the right balance—harnessing AI’s benefits for patients while protecting patients’ rights and maintaining trust—will require ongoing dialogue between clinicians, AI developers, legal experts, and policymakers. The pathway forward likely involves shared accountability, robust validation of AI tools, transparency in algorithm performance, and perhaps new insurance or no-fault compensation mechanisms for when even a well-intentioned AI causes harm [39]. By anticipating these medico-legal issues and addressing them head-on, the field can ensure that generative AI in breast imaging is implemented in a manner that is not only innovative and effective, but also ethical and legally sound.

## 5. Challenges and Limitations

Despite their potential, the use of synthetic data presents several challenges, summarized in Table 3. The primary concern intuitively relates to the reliability of the images generated. Although generative AI offers great potential, it also presents notable limitations—chief among them is the risk that models trained on biased data may reproduce and even intensify these biases, potentially resulting in inaccurate predictions, particularly for underrepresented populations [5].

An additional significant limitation is that several studies have utilized enriched datasets, which include a higher proportion of true positive cases than would be expected in a routine screening population. Although these datasets enhance statistical power and are more convenient, they fail to represent the typical prevalence and spectrum of breast cancer.

Consequently, common elements of study design may introduce bias, potentially leading to overestimation or underestimation of diagnostic accuracy [40,41].

Moreover, current generative models may not perform uniformly across all breast cancer phenotypes. Subtle microcalcification clusters and ductal carcinoma in situ, complex architectural distortions, non-mass enhancement patterns (typically characterized on MRI), and rare histological subtypes remain underrepresented in most training corpora. As a result, synthetic images in these domains may either be poorly realistic or systematically biased, with a risk of masking precisely those lesions that are already challenging in real-world practice. Future work should therefore report performance and perceived realism stratified by lesion type and imaging phenotype.

Throughout this review, we refer to “high-quality synthetic mammograms” in a pragmatic sense, based on three recurring criteria in the literature: (i) reader studies in which radiologists are unable, or only marginally able, to distinguish synthetic from real mammograms; (ii) statistical analyses demonstrating close alignment of pixel-level and texture distributions between real and synthetic images; and (iii) evidence that the inclusion of synthetic images in training improves task performance without introducing visually obvious artifacts.

Additionally, according to Ahn JS et al., recent clinical validation efforts, especially those examining treatment outcomes, are frequently limited by retrospective study designs that may introduce unforeseen biases. This highlights the importance of conducting prospective studies, which are essential to thoroughly evaluate the effectiveness and reliability of AI tools in clinical practice [42].

Furthermore, standardized validation protocols for synthetic medical images are lacking. Rigorous, task-specific validation approaches are necessary to determine whether the use of synthetic data truly leads to performance improvements. In addition, regulatory uncertainty represents a significant barrier to the clinical integration of synthetic data. Regulatory agencies have yet to establish detailed guidelines on the generation, validation, and documentation of synthetic data, particularly in high-risk domains such as medicine. The FDA’s 2021 action plan for AI/ML in medical devices does not yet define synthetic data-specific validation. Ethical and regulatory challenges surrounding Generative AI in healthcare include the need for clear guidelines from bodies like the FDA and EMA to ensure synthetic data complies with safety and privacy standards. There is a risk of introducing or amplifying bias, which calls for robust methods to detect and correct it, ensuring equity across diverse populations. Regulations may also evolve to require patient consent for using anonymized data in synthetic generation [5].

For clinical institutions, validation of synthetic datasets before deployment in machine-learning pipelines should follow a structured approach. First, governance and privacy committees should review how the generative model was trained and how source data were handled. Second, statistical comparisons (e.g., distribution of breast density, lesion types, intensity histograms) should verify that synthetic images are broadly consistent with the local population. Third, models should be trained with and without synthetic augmentation and evaluated on independent real-world test sets, with particular attention to performance changes. Finally, subgroup analyses across age, breast density, and other relevant demographics are needed to ensure that synthetic augmentation does not degrade performance in vulnerable groups.

Thus, in summary, the absence of standardized validation protocols for synthetic medical images makes it challenging to accurately assess their true contribution to model performance. Rigorous, task-specific validation and algorithm testing are essential not only to evaluate diagnostic utility but also to ensure interpretability and generalizability of results. Moreover, regulatory approval remains a major barrier, particularly when algorithms are intended to support clinical decision-making. Regulatory bodies, such as the FDA, may require robust clinical trials to demonstrate the safety and efficacy of these tools—a process that is both time-consuming and costly [43]. To provide a structured synthesis of the key issues discussed, Table 4 shows a gap analysis highlighting the main problems currently limiting the use of synthetic data and generative AI in breast imaging, the present state of evidence, and potential future directions to address these challenges.

## 6. Future Directions

The evolution of generative models—such as conditional GANs, advanced diffusion models, and transformer-based approaches—is expected to improve the quality, controllability, and scalability of synthetic images. However, these technological advancements raise fundamental questions regarding the role of machines in clinical decision-making processes. As discussed in the study “The Picasso’s skepticism on computer science and the dawn of generative AI,” the progress of generative artificial intelligence necessitates careful consideration of maintaining the so-called “human-in-the-loop,” emphasizing the need to retain human judgment within automated systems. The authors symbolically invoke Picasso’s skepticism toward technology to highlight the risk that, in pursuit of efficiency and scalability, the critical, interpretative, and relational capacities that characterize human intervention—particularly in high-stakes domains such as oncological diagnosis—may be lost [1].

Concurrently, there is an urgent need for standardized protocols and regulatory frameworks. Scientific societies and regulatory authorities must play a central role in defining technical and ethical criteria for the use of synthetic data, ensuring that they contribute to safe, reproducible, and accessible healthcare. Within this context, the approach suggested by the study advocates against fully delegating critical tasks to machines, instead promoting the development of collaborative models where artificial intelligence acts as a support rather than a substitute for clinical responsibility [28].

Finally, for widespread clinical adoption, several technical and ethical barriers remain pivotal. Technically, the field still lacks standardized, task-specific protocols to validate whether synthetic images truly improve model performance and generalizability, and there is a persistent risk of propagating or amplifying existing biases and of memorizing training data. Ethically and legally, questions regarding consent for using real images to train generative models, transparency in disclosing the role of synthetic data, and robust provenance and tamper-detection mechanisms are not yet fully resolved. Addressing these issues is a prerequisite for responsible integration of synthetic data into routine breast imaging practice.

## 7. Conclusions

Synthetic data and generative AI represent transformative advancements in breast imaging, offering solutions to longstanding limitations such as data scarcity, privacy concerns, and biased training datasets. These technologies enable the creation of diverse, realistic mammographic images, which enhance the generalizability and robustness of AI algorithms, streamline radiologist training, and foster more equitable clinical outcomes. Nonetheless, the implementation of synthetic data in clinical environments is not without challenges. Concerns around data authenticity, potential introduction of biases, and the lack of established validation protocols underscore the necessity for rigorous, task-specific assessment methodologies.

Moreover, the expanding use of generative AI raises critical medico-legal questions, particularly around liability, professional accountability, and the integrity of medical images. Proactive engagement with these issues through robust regulatory frameworks, ethical guidelines, and clearly defined accountability models is essential. The pathway forward involves fostering multidisciplinary collaborations among radiologists, AI developers, policymakers, legal experts, and patient representatives. This collaborative approach is crucial to ensure that generative AI technologies are ethically sound, legally robust, clinically valuable, and aligned with the broader objectives of personalized, equitable, and effective breast cancer screening and diagnosis.

## Figures and Tables

**Table 1:** Different methods of generative AI.

Method	Description	Applications in Breast Imaging	Advantages	Limitations	Validation Needs
Generative Adversarial Networks (GANs)	Generator and discriminator compete to create realistic images.	Mammographic density transformation, lesion synthesis, dataset augmentation, contralateral breast modeling.	High realism, diverse image generation, ability to simulate rare findings.	Risk of bias amplification, mode collapse, high computational demand.	Multi-reader validation studies, detection of GAN-specific artifacts, equity assessment.
Diffusion Models	Iteratively transform noise into structured images conditioned on prompts.	Controlled synthesis of mammograms with defined features (density, lesion morphology).	High fidelity, controllability of attributes, effective for targeted synthesis.	Require large datasets and training resources, still early adoption in medical imaging.	Benchmarking against real cases, blinded radiologist assessment, standardized fidelity metrics.
Transformer-based Generative Models	Use self-attention to capture long-range dependencies in imaging data.	Emerging use for high-resolution mammogram synthesis and report-to-image generation.	Handle complex global structures, scalable to large datasets.	Limited breast imaging studies to date, interpretability challenges.	Prospective testing in radiology workflows, standardized interpretability reporting.

**Table 2:** Open research questions.

Question	Rationale	Clinical/Regulatory Relevance
How can synthetic data be validated for clinical equivalence to real images?	Ensures diagnostic accuracy and builds clinician trust.	Required for regulatory approval (FDA, EMA) and clinical adoption.
What strategies best mitigate bias in generative models?	Reduces health disparities and improves generalizability.	Critical for equitable screening programs and avoiding algorithmic discrimination.
How can regulatory and ethical frameworks be standardized?	Facilitates safe and transparent integration of synthetic data.	ESGAR consensus (2024) and AUR tutorial (2024) highlight urgent need for harmonized protocols.
What is the role of synthetic data in radiology education and simulation?	Supports training with rare cases and diverse presentations.	Emerging consensus that standardized validation is necessary before routine use in curricula.
Question		Why
How can synthetic data be validated for clinical equivalence to real images?		Ensures diagnostic accuracy and builds clinician trust for clinical adoption.
What strategies best mitigate bias in generative models for diverse populations?		Reduces health disparities and improves generalizability of AI tools.
How can regulatory and ethical frameworks be standardized for synthetic data use?		Facilitates safe, transparent, and widespread clinical integration.

**Table 3:** Challenges and potential solutions in using synthetic images in clinical practice.

Challenge	Explanation	Potential Solutions
Reliability of Synthetic Images	Concerns about clinical equivalence and accuracy.	Rigorous clinical validation against real datasets.
Dataset Biases	Synthetic data might amplify existing biases.	Diverse and balanced training datasets, fairness algorithms.
Regulatory and Ethical Uncertainty	Lack of standardized guidelines for clinical integration.	Collaboration with regulatory bodies to establish clear frameworks.
Image Authenticity and Integrity	Risk of falsified medical images.	Implementation of cryptographic signatures and blockchain technology.

**Table 4:** Gap Analysis in Synthetic Data and Generative AI for Breast Imaging.

Problem	Current Situation	Future Directions
Data scarcity & bias	Limited real-world datasets, skewed by demographics and density	Use of synthetic augmentation, fairness audits, global consortia
Validation standards	No uniform protocols; reliance on enriched datasets	Adoption of ESGAR/AUR guidelines, task-specific validation, prospective trials
Medico-legal accountability	Liability unclear; few case precedents	Shared accountability models, regulatory clarity, provenance tracking
Image authenticity	Risk of deepfakes, tampering	Cryptographic watermarking, blockchain provenance, regulatory oversight
Clinical integration	Pilot use, no large-scale adoption	Prospective multi-centre validation, transparent reporting, regulatory harmonization

## Data Availability

No new data were created or analyzed in this study. Data sharing is not applicable to this article.
